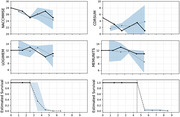# Artificial Intelligence (AI)‐based dynamic predictions of longitudinal and survival data of Alzheimer's disease

**DOI:** 10.1002/alz70856_103788

**Published:** 2025-12-24

**Authors:** Sori Kim Lundin, Yong Chen, Paul E Schulz, Cui Tao

**Affiliations:** ^1^ Center for Biomedical Semantics and Data Intelligence (CBSDI), University of Texas Health Science Center at Houston, Houston, TX, USA; ^2^ School of Public Health, The University of Texas Health Science Center at Houston, Houston, TX, USA; ^3^ Perelman School of Medicine, University of Pennsylvania, Philadelphia, PA, USA; ^4^ John P. and Kathrine G. McGovern Medical School at UTHealth, Houston, TX, USA; ^5^ Mayo Clinic Department of Artificial Intelligence and Informatics, Jacksonville, FL, USA

## Abstract

**Background:**

Dynamically predicting patients at risk of Alzheimer's disease (AD) is crucial for timely treatment and care in precision medicine.

**Method:**

We present a novel architecture, a Kolmogorov‐Arnold networks (KANs)‐based joint prediction model of longitudinal and survival data (JM‐KAN). Real‐life data of patients with Mild Cognitive Impairment (MCI) with no prior AD diagnosis from National Alzheimer's Coordinating Center (NACC) dataset was utilized for individualized predictions. We built joint models with landmark times every 1 year up to 4 years after the MCI diagnosis, and predicted survival outcomes one year after each landmark (i.e. 1, 2, 3, and 4 years). Integrated AUC (iAUC), and integrated Brier score (iBS) were measured for existing prediction models and our novel architecture.

**Result:**

Out of 2711 unique patients with MCI, 821 patients progressed to develop AD. Mean time‐to‐event in years was 3.19 (standard deviation (SD): 2.29). The number of visits prior to AD diagnosis was 4.03 (SD: 2.41). With iAUC of 0.789, iBS of 0.118, JM‐KAN has both the highest iAUC and lowest iBS among joint models, which indicates superiority in discrimination and overall performance. For individualized predictions, the model successfully predicted both longitudinal risk factors and survival probability, as new information came in every year.

**Conclusion:**

Using neural network‐based Artificial Intelligence (AI) model, we demonstrated that this architecture improves upon prior methods in dynamically updating predictions of AD as new longitudinal data becomes available.